# Host Plants Shape the Codon Usage Pattern of Turnip Mosaic Virus

**DOI:** 10.3390/v14102267

**Published:** 2022-10-15

**Authors:** Lang Qin, Shiwen Ding, Zhilei Wang, Runzhou Jiang, Zhen He

**Affiliations:** 1 College of Plant Protection, Yangzhou University, Wenhui East Road No.48, Yangzhou 225009, China; 2 Joint International Research Laboratory of Agriculture and Agri-Product Safety of Ministry of Education of China, Yangzhou University, Yangzhou 225009, China

**Keywords:** turnip mosaic virus, codon usage, composition bias, natural selection, host adaptation

## Abstract

Turnip mosaic virus (TuMV), an important pathogen that causes mosaic diseases in vegetable crops worldwide, belongs to the genus *Potyvirus* of the family *Potyviridae*. Previously, the areas of genetic variation, population structure, timescale, and migration of TuMV have been well studied. However, the codon usage pattern and host adaptation analysis of TuMV is unclear. Here, compositional bias and codon usage of TuMV were performed using 184 non-recombinant sequences. We found a relatively stable change existed in genomic composition and a slightly lower codon usage choice displayed in TuMV protein-coding sequences. Statistical analysis presented that the codon usage patterns of TuMV protein-coding sequences were mainly affected by natural selection and mutation pressure, and natural selection was the key influencing factor. The codon adaptation index (CAI) and relative codon deoptimization index (RCDI) revealed that TuMV genes were strongly adapted to *Brassica oleracea* from the present data. Similarity index (SiD) analysis also indicated that *B*. *oleracea* is potentially the preferred host of TuMV. Our study provides the first insights for assessing the codon usage bias of TuMV based on complete genomes and will provide better advice for future research on TuMV origins and evolution patterns.

## 1. Introduction

Turnip mosaic virus (TuMV) belongs to one of the largest genera of plant RNA viruses, namely, *Potyvirus*, which is in the family *Potyviridae* [1] TuMV is known to infect a wide range of plant species that mostly belong to the family *Brassicaceae* [2]. In nature, TuMV can be transmitted by aphids in a non-persistent manner. The size of TuMV virions is approximately 720 nm × 15~20 nm, and they are composed of 95% coat protein and 5% RNA. TuMV virions contain a positive single-stranded RNA molecule, which is approximately 9830 nucleotides (nts) in length. The five ends of the TuMV genome are covalently linked to a viral coding protein (VPg). The genome has a main open reading frame (ORF) encoding a large polyprotein and non-translated regions on each end of the molecule. Under the action of viral encoded proteases, a total of 10 functional proteins are obtained after the proteolytic process of the polyprotein, such as the first protein (P1; the molecular weight is 40 kDa), helper component protease (HC-Pro; 52 kDa), protein 3 (P3; 40 kDa), the first 6 kDa protein (6K1; 6 kDa), cylindrical inclusion body protein (CI; 72 kDa), the second 6 kDa protein (6K2; 6 kDa), encoding viral genome-related protein (VPg; 22 kDa), small nuclear inclusion body a (NIa; 27 kDa), small nuclear inclusion body b (NIb; 60 kDa), and coat protein (CP; 33 kDa) [1]. A small, overlapping ORF encodes a truncated frameshift product, namely, PIPO protein [3].

Normally, the genetic code allows 61 triplet codons to encode 20 amino acids, and codons encoding the same amino acid are termed synonymous codons [4,5]. Intriguingly, synonymous codons are not randomly used, the synonymous codons are also not used equally in various organisms or even in different gene groups of the same genome, creating a bias in codon usage, which is known as codon usage bias (CUB) [6,7,8,9]. Codon usage patterns are influenced by many factors, such as mutation pressure, compositional constraints, natural selection, gene length, replication, hydrophobicity, selective transcription, gene function, secondary protein structure, and the external environment [4,6,8,9,10,11,12,13,14]. Evolution, adaption, overall viral fitness, and evasion of host cell responses and survival are affected by the codon usage bias of viruses and their hosts. Due to their low codon usage bias, most RNA viruses can reduce their level of competition with host genes and thereby effectively replicate in host cells.

As one of the most-studied plant-infecting RNA viruses in the area of evolution, the genetic variation, population structure, evolutionary rate, timescale, and migration of TuMV isolated from *Raphanus sativus*, *Brassica oleracea*, *Brassica juncea*, *Brassica rapa*, *Sisymbrium loeseli*, and *Rapistrum rugosum* in Belgium, China, Greece, Germany, South Korea, Japan, Iran, Turkey, UK, Poland, Russia, USA, Slovakia, Mexico, Italy, and Brazil have been stated based on analyses of partial or complete genome sequences [15,16,17,18,19,20,21]. To date (August 2022), one hundred and eighty-four genomic sequences of TuMV non-recombinant isolates from South Korea, China, Iran, Turkey, the UK, Poland, Russia, Greece, Belgium, Germany, Italy, The Czech Republic, Australia, and Brazil have been reported [22,23]. However, the synonymous codon usage pattern of TuMV is still not fully reported.

In the present study, we conducted detailed codon usage analyses of TuMV non-recombinant isolates based on 184 genomic sequences to assess the evolutionary adaptation of this virus to its hosts. We explored the factors that shape the codon usage patterns of TuMV and provided a new perspective on the genetic divergence of TuMV. To the best of our knowledge, this study provides the first insights into the codon usage patterns of TuMV.

## 2. Materials and Methods

### 2.1. Virus Isolates

One hundred and eighty-four genomic sequences of TuMV non-recombinant isolates were retrieved from GenBank [22,23]. The details of those isolates, such as geographical location, date of collection, and host, are shown in Table S1.

### 2.2. Recombination and Phylogenetic Analysis

All of the TuMV sequences that are described in Table S1 were aligned using CLUSTAL X2 [24]. Putative recombination incidents of aligned TuMV were identified by several methods within the RDP4 software package [25], such as the GENECONV, RDP, BOOTSCAN, 3SEQ, CHIMAERA, MAXCHI, and SISCAN programs [26,27,28,29,30,31,32]. Through a phylogenetic approach in the RDP4 package, parent/donor assignments were proved. There were at least four different methods (*p*-value of <1.0 ×10^−6^) in the RDP4 package that supported the putative recombinants. These analyses were performed by the different detection programs using the default settings.

We used the neighbor-joining (NJ) method in MEGA v11 [33] to assess the phylogenetic relationships of the polyprotein-coding sequences of TuMV. The NJ analyses used were evaluated by Kimura’s two-parameter with 1000 bootstrap replicates [34]. The inferred trees were displayed using TreeView [35].

### 2.3. Nucleotide Composition Analysis

The nucleotide composition of TuMV polyprotein and the 11 protein-coding sequences were calculated after removing five non-bias codons, such as UGA, UAG, and UAA (termination codons) and UGG and AUG (the only codons encoding Trp and Met, respectively). The component parameters of the TuMV polyprotein and the 11 protein-coding sequences were then calculated. The entire nucleotide composition (e.g., A, C, U, and G%) and the total AU and GC contents were calculated using BioEdit version 5.0.9 [16]. CodonW 1.4.2 package was used for the analysis of the nucleotide composition at the third codon position of the TuMV coding sequences (e.g., A3, C3, U3, and G3%). The GC contents of the first base of codon (GC1), the second base, and the third base of codon (GC2, GC3) were employed for analysis in an online program (http://www.bioinformatics.nl/emboss-explorer/ (accessed on 25 August 2022)), where GC12 is the mean of GC1 and GC2.

### 2.4. Relative Synonymous Codon Usage (RSCU) Analysis

The RSCU value of a codon is the ratio between the observed and expected usage frequencies [36]. The RSCU values were calculated using the following formula:

(1) $$RSCU_{ij}=\frac{g_{ij}}{\sum_{j}^{ni}g_{ij}}\times ni\;$$

In this formula, $RSCU_{ij}$ is the value of the i-th codon for the j-th amino acid, the $g_{ij}$ is the observed number of the i-th codon for the j-th amino acid, and “ni” kind of represents the degenerate numbers of synonymous codons which encode the j-th amino acid. An RSCU value of 1 suggests no bias for the codon. While codons with RSCU values <0.6 and >1.6 are defined as low and high-frequency codons, respectively. MEGA v11 software was used to calculate the RSCU values of the TuMV polyprotein and the 11 protein-coding sequences [33]. The available coding sequences of *R. sativus, B. rapa, B. oleracea,* and *R. juncea* were downloaded from the GenBank database. The host RSCU values were calculated using MEGA v11 software [33].

### 2.5. Principal Component Analysis (PCA)

A multivariate statistical method called PCA was used to identify the correlations between variables and samples. After removing the three termination codons and UGG and AUG codons, a 59-dimensional vector was used to represent each strain of the 12 data sets where different dimensions corresponded to each sense codon’s RSCU value. PCA analysis was used by Origin 8.0.

### 2.6. Effective Number of Codons Analysis (ENC)

The ENC values, which were calculated using CodonW v1.4.2 software and indicate the degree of codon usage bias, ranged from 20 (an extreme codon usage bias for which only one synonymous codon was used) to 61 (no bias, the synonymous codons were equally used) [37]. The ENC values were calculated as:

(2) $$ENC=2+\frac{9}{{\bar{F}}_{2}}+\frac{1}{{\bar{F}}_{3}}+\frac{5}{{\bar{F}}_{4}}+\frac{3}{{\bar{F}}_{6}}\;$$

where ${\bar{F}}_{k}$ (*k* = 2, 3, 4, 6) is on behalf of the average of $F_{k}$*,* and ***k*** indicates the k-fold degenerate amino acids. $F_{k}$ is estimated as follows:

(3) $$F_{k}=\frac{nS-1}{n-1}\;$$

where n is the total number of the observed values of the codon for the corresponding amino acid and

(4) $$S=\overset{k}{\underset{i=1}{\sum }}{\left(\frac{n_{i}}{n}\right)}^{2}\;$$

where $n_{i}$ stands for the total number of the i-th codon for that amino acid.

The ENC analysis is used to measure the absolute codon usage bias of the TuMV genes. Typically, a gene with ENC values ≤ 35 indicates significant CUB. It is considered that smaller ENC values show stronger CUB.

### 2.7. ENC-Plot Analysis

ENC-plot analysis (with the GC3s value on the horizontal ordinate and the ENC value on the longitudinal coordinate) was used to provide the role of mutation pressure in codon usage bias. When mutation pressure is the only factor, the dot lies on or around the standard curve. Otherwise, it is influenced by selection and other factors. The expected ENC was conducted as:

(5) $$ENC\;\mathrm{expected}=2+s+\left(\frac{29}{s^{2}+{\left(1-s\right)}^{2}}\right)\;$$

where ***s*** represents the value of GC3s.

### 2.8. Parity Rule 2 Analysis (PR2)

Applying PR2 bias plots to investigate the influence of natural selection and mutation pressure on the codon usage of the TuMV. The value of AU-bias (A3/(A3 + U3) as the ordinate against GC-bias G3/(G3 + C3) as the abscissa), respectively. The center of the plot is 0.5, which indicates a balance between mutation pressure and natural selection.

### 2.9. Neutrality Analysis

In the neutrality plot graph, GC12 and GC3 are shown as the ordinate and abscissa, respectively. The mutational force is represented by the slope of the regression line between GC12 and GC3 contents. If there is no selection pressure or the selection pressure is weak, the slope of the regression line is near 1.0. Conversely, if the regression line slope deviates from 1.0, indicating that natural selection has a key role in codon bias.

### 2.10. Codon Adaptation Index (CAI) Analysis

The CAI analysis was computed by a web server (http://genomes.urv.cat/CAIcal/RCDI/ (accessed on 25 August 2022)) and was used to predict the adaptation of individual TuMV genes to their potential host. Normally, higher CAI values (e.g., from 0 to 1) indicate stronger adaptability to the host. Due to the lack of relevant codon usage data of the hosts *R. rugosum* and *S. loeselii*, the CAI analysis was performed by the remaining four hosts.

### 2.11. Relative Codon Deoptimization Index (RCDI) Analysis

The RCDI values are calculated using an online program (http://genomes.urv.cat/CAIcal/RCDI/ (accessed on 25 August 2022)) for the TuMV polyprotein, and the 11 protein-coding sequences were used to identify trends in codon deoptimization. If the RCDI values were equal to 1, this indicated that the virus displayed a host-adapted codon usage pattern. Conversely, RCDI values higher than 1 indicate lower adaptability due to the lack of relevant codon usage data of the hosts *R. rugosum* and *S. loeselii*. The RCDI analysis was performed by the remaining four hosts.

### 2.12. Similarity Index (SiD) Analysis

SiD analysis is a widely used method for determining the effect of the codon usage bias of hosts. The SiD value was calculated as:

(6) $$R\left(A,B\right)=\frac{\sum_{i=1}^{59}a_{i}b_{i}}{\sqrt{\sum_{i=1}^{59}b_{i}^{2}\;\sum_{i=1}^{59}a_{i}^{2}}}$$

(7) $$D\left(A,B\right)=\frac{1-R\left(A,B\right)}{2}$$

where ***a_i_*** represents the RSCU values of 59 synonymous codons of the TuMV coding sequences, and ***b_i_*** represents the RSCU values of identical codons of the host. The potential impact of the host’s entire codon usage on the different clades of the TuMV gene is represented by the SiD [$D\left(A,B\right)$] (from 0 to 1.0). Higher values generally indicate that the host plays a significant role in codon usage.

## 3. Results

### 3.1. Recombination and Phylogenetic Analysis

Generally, recombination can influence the topology of phylogenetic trees and overall codon usage patterns regardless of gene or genome levels [38,39]. A total of 184 TuMV non-recombinant coding sequences [22,23] from *B. juncea*, *B. oleracea*, *B. rapa*, *R. sativus*, *R. rugosum*, and *S. loeselii* were used in the following phylogenetic and codon usage analyses.

Phylogenetic analyses were conducted using the NJ methods based on the complete polyprotein of TuMV. The NJ trees that are based on complete polyprotein sequences are shown (Figure S1). Six lineages with certain degrees of host origins were formed based on the complete polyprotein-coding sequences (Figure S1). As Yasaka et al. (2017), Kawakubo et al. (2021), and Kawakubo et al. (2022) reported, these six major genetic groups that are based on polyprotein-coding sequences were clustered into the Orchis, Asian-BR, basal-B, basal-BR, Iranian, and world-B groups.

### 3.2. Nucleotide Bias Analysis

The nucleotide compositions of the complete polyprotein and 11 protein-coding sequences of TuMV were assessed to explore the effect of compositional constraints on codon usage. For the polyprotein, nucleotides A and G were most abundant, with mean compositions of 32.09 ± 0.55% and 24.21 ± 0.51% (Table S2), respectively, and were followed by U (22.55 ± 0.38%) and C (21.15 ± 0.45%). Similarly, for the individual protein-coding sequences, we also found that the nucleotides A and G were most abundant in the P1, HC-Pro, 6K2, VPg, NIb, CP, and PIPO coding regions (Table S2), while nucleotides A and U were rich in P3, CI, and NIa coding regions. The nucleotides A and C were most abundant in the 6K1 (Table S2). However, the third position’s nucleotide composition of synonymous codons (e.g., A3S, U3S, G3S, and C3S) was inconsistent with the nucleotide composition at the complete polyprotein level. The most frequent nucleotide was A3S (39.55 ± 1.78%), which was followed by C3S (30.82 ± 1.35%), U3S (28.96 ± 1.21%), and G3S (29.03 ± 1.70%) (Table S2). For the protein-coding sequences, the A3S and G3S were only found to be most abundant in the coding sequences of the CP, Nib, and PIPO coding regions (Table S2); compared with the nucleotides A3S and U3S, which were most abundant in the P3 and CI coding region sequences, the nucleotides A3S and C3S were most abundant in the 6K1, 6K2, HC-Pro, NIa, P1, and VPg coding sequences (Table S2). The composition of AG is better than the UC of complete polyproteins and 11 protein-coding sequences (Table S2), indicating that there is an AG-rich composition for the TuMV coding sequences.

### 3.3. Relative Synonymous Codon Usage Analysis of TuMV and Its Hosts

RSCU analysis was conducted to estimate the codon usage patterns of TuMV polyprotein and 11 protein-coding sequences. Thirteen of the 18 preferred codons were A/C (A-ended 7, C-ended 6) in the complete polyprotein-coding region (Table 1). A/C-ended codons were also preferred in individual protein-coding sequences, P1 (A-ended 5, C-ended 6, HC-Pro (A-ended 8, C-ended 5), 6K2 (A-ended 5, C-ended 7), Vpg (A-ended 6, C-ended 6), NIa (A-ended 7, C-ended 5), and PIPO (A-ended 8, C-ended 5), except P3 (A-ended 6, U -ended 5), 6K1 (A-ended 6, U -ended 6), CI (A-ended 8, U-ended 4) and CP (C-ended 5, G-ended 5) (Table 1). The results show that the A/C-terminal codon is slightly popular in the TuMV coding sequence. Among these preferred codons in the complete polyprotein-coding region, the RSCU values of four codons were >1.6, and the highest value was for CCA (2.36), indicating extreme overrepresentation, and the remaining preferred codons had RSCU values >0.6 and <1.6. Additionally, to determine the potential influences of hosts on the codon usage patterns of the TuMV isolates, the RSCU patterns of the TuMV polyprotein-coding sequences were correlated with those of *B. juncea*, *B. oleracea*, *B. rapa*, *R. sativus*, *R. rugosum*, and *S. loeselii.* Seventeen of the 18 preferred codons were A/U -ended (A-ended: 5; U-ended: 12) for *B. juncea*, and *R. rugosum* had a similar pattern of use, with A-ended: 6, U-ended: 12. Whereas the four hosts *B. oleracea*, *B. rapa*, *R. sativus*, and *S. loeselii* had almost the same codon usage pattern, 13 of the 18 preferred codons were C/U-ended (C-ended: 4; U-ended: 9) (Table S3). Overall, a mixture of antagonism and coincidence was discovered in the codon usage patterns of TuMV and its six hosts based on polyprotein-coding sequences (Table S3).

### 3.4. Trends in Codon Usage Variations

**Figure 1:**
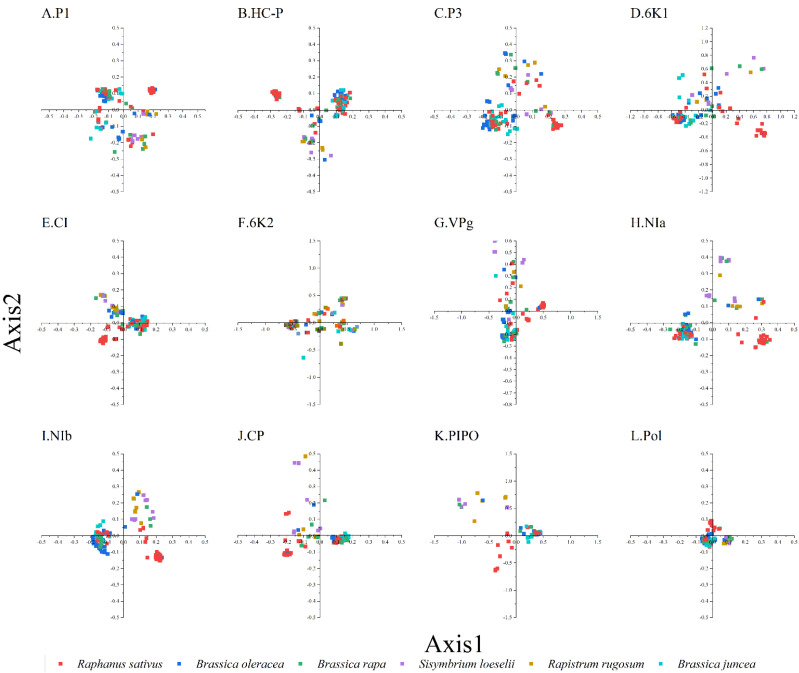
Principal component analysis (PCA) is based on the relative synonymous codon usage (RSCU) values of the 59 synonymous codons for the individual protein (**A**–**K**), complete polyprotein (**L**), and coding sequences of turnip mosaic virus. The *Raphanus sativus*, *Brassica oleracea*, *Brassica rapa*, *Sisymbrium loeseli*, *Rapistrum rugosum* and *Brassica juncea* hosts are represented in red, blue, green, purple, orange, wathet blue and brown, respectively.

To study the synonymous codon usage variations in the coding sequences of TuMV, principal component analysis was used. The first four axes (axes 1–4) of the complete polyprotein and individual protein-coding sequences were recorded for more than 60% of the variation (Figure S2). In addition, it can be seen from the figure that axis 1 is the key factor that affects the codon usage for the TuMV coding regions (Figure 1). Moreover, based on the RSCU values on the first two axes, we discovered the distribution of the complete polyprotein and 11 protein-coding sequences in different hosts (Figure S2). We found obvious an overlap between the different hosts in the PCA analysis of TuMV complete polyprotein and individual protein-coding regions, which suggests distinct codon usage trends (Figure 1).

### 3.5. Codon Usage Bias of TuMV

**Figure 2:**
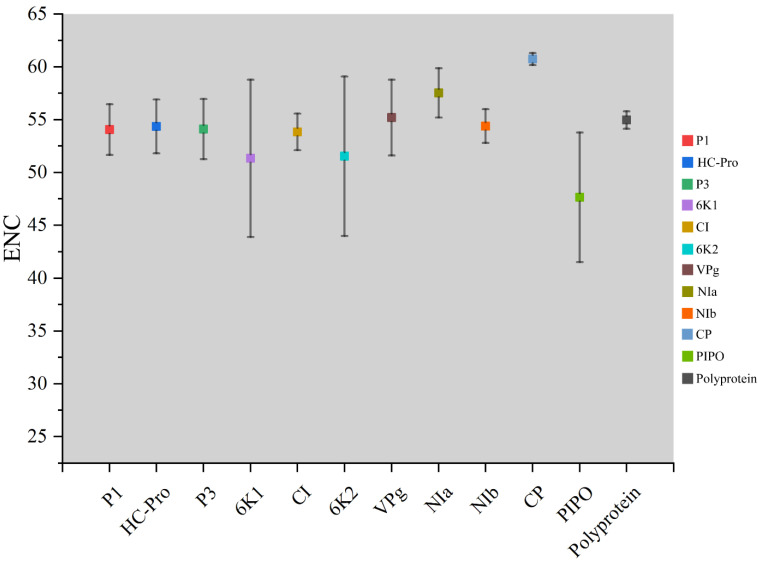
Effective number of codons analysis (ENC) values for the eleven protein and complete polyprotein-coding sequences of turnip mosaic virus.

The ENC values were calculated to show the magnitude of the choice of TuMV genome codon usage. Individually, maximum ENC values were observed for the CP coding sequences, while minimum values were found in the PIPO coding sequences (Figure 2). Polyprotein and 10 coding sequences of TuMV, the average ENC values were all more than 45 (Table S2) (Figure 2). These results suggested that there was a relatively conserved nucleotide composition with slightly lower codon usage choice in the TuMV coding sequences.

### 3.6. ENC-Plot Analysis

**Figure 3:**
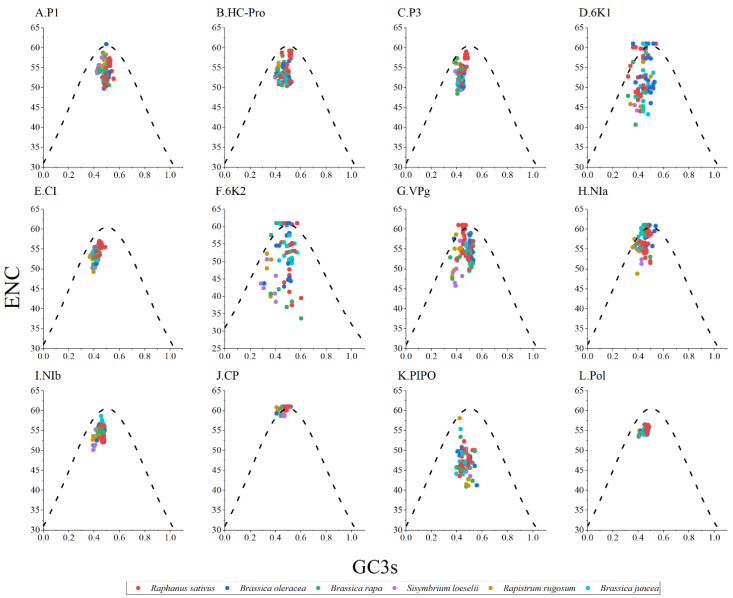
Effective number of codons analysis (ENC)-plot analysis of the individual protein (**A**–**K**) and complete polyprotein (**L**) coding sequences of turnip mosaic virus, with ENC against the GC3s of different hosts. The black dotted line represents the standard curve when the codon usage bias is determined by only the GC3s composition. The *Raphanus sativus*, *Brassica oleracea*, *Brassica rapa*, *Sisymbrium loeseli*, *Rapistrum rugosum* and *Brassica juncea* hosts are represented in red, blue, green, purple, orange, wathet blue and brown, respectively.

ENC-GC3s plot analysis was performed to study the forces that influenced the codon usage bias of the TuMV protein-coding regions. Generally, if the points fall below the expected curve, it means that the codon usage is more strongly affected by natural selection pressure. However, mutation pressure is indicated when the data points fall on the expected curve. As shown in Figure 3, the TuMV isolates from different hosts typically cluster below the expected curve; it implies that natural selection dominated over mutation pressure, while the influence of mutation was not completely absent (Figure 3).

### 3.7. Neutrality Plot

**Figure 4:**
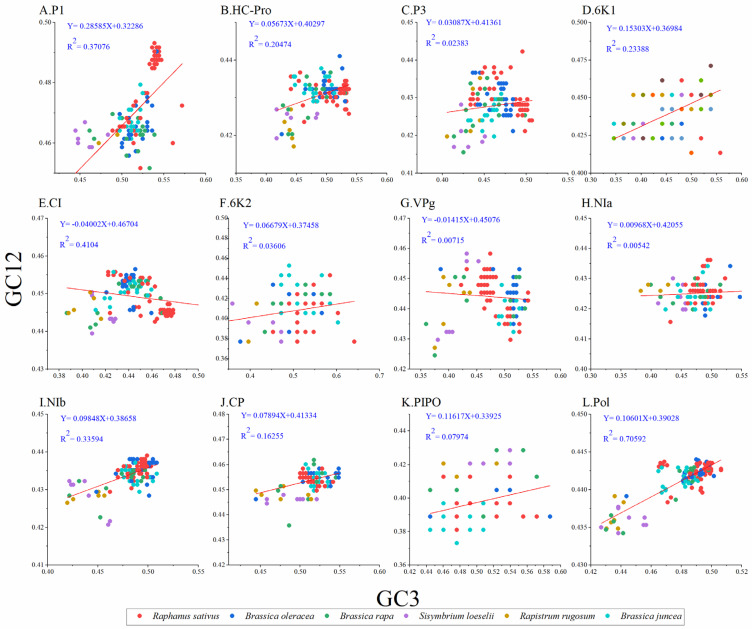
Neutrality plot analysis of GC3s against GC12s for the individual protein (**A**–**K**) and complete polyprotein (**L**) coding sequences of turnip mosaic virus. The *Raphanus sativus*, *Brassica oleracea*, *Brassica rapa*, *Sisymbrium loeseli*, *Rapistrum rugosum* and *Brassica juncea* hosts are represented in red, blue, green, purple, orange, wathet blue and brown, respectively.

To unravel the extent of influence between mutation pressure and natural selection on codon usage in TuMV, we performed a neutrality analysis between GC12 and GC3. Normally, nucleotide changes at the third position of the codon do not result in amino acid changes, which are considered to reflect only a mutational force. Whereas, if a nucleotide change produces a change in the amino acid, it is considered a mutation pressure. Among the protein-coding sequences of TuMV, significant positive correlations were observed between the GC12 and GC3 values for the TuMV polyprotein (Figure 4) and the P1, HC-Pro, P3, 6K1, 6K2, NIa, NIb, PIPO, and CP coding sequences (Figure 4A, B, C, D, F, H, I, J, and K respectively); in contrast, the GC12 and GC3 values for the TuMV CI and VPg coding sequences (Figure 4G) showed no significant correlations. The slope of the linear regression for the polyprotein-coding sequences was 0.106 (Figure 4), indicating that mutation pressure accounted for 10.6% of the pressure on codon usage, while natural selection accounted for 89.4% of the pressure. All of these results showed that natural selection was the principal force driving the TuMV coding sequences’ codon usage bias.

### 3.8. Parity Analysis

**Figure 5:**
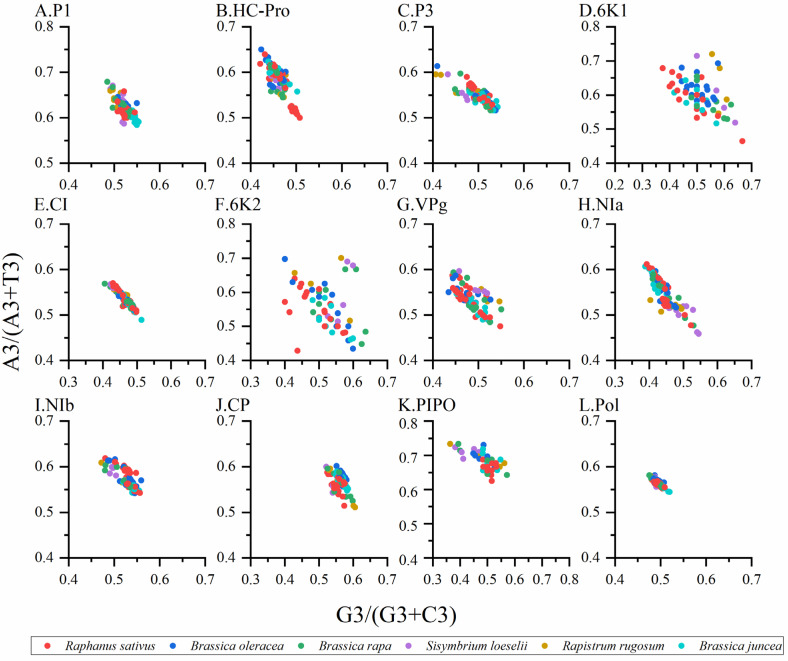
Parity plot showing the presence of AT bias [A3%/(A3% + T3%)] and GC bias [G3%/(G3% + C3%)] for the individual protein (**A**–**K**) and complete polyprotein (**L**) coding sequences of turnip mosaic virus. The center of the plot, where the value of both the coordinates is 0.5, indicates the place where there is no bias in the mutation or selection rates. The *Raphanus sativus*, *Brassica oleracea*, *Brassica rapa*, *Sisymbrium loeseli*, *Rapistrum rugosum* and *Brassica juncea* hosts are represented in red, blue, green, purple, orange, wathet blue and brown, respectively.

Normally, when PR2 biases at the third codon position are plotted in four-codon sequences of individual genes, it is considered that the PR2 plots are especially useful. Therefore, we constructed PR2 plots to confirm the influence of mutation pressure and natural selection on the CUB. When the plot lies in the center (e.g., A = U and G = C), both coordinates are 0.5, and no bias is present in the selection or mutation pressure [6]. The results showed that nucleotides A was more frequently used than U, while nucleotides G and C were used at similar frequencies in the TuMV coding sequences (Figure 5A–L), which indicated that the codon usage bias of TuMV was also shaped by natural selection and other factors.

### 3.9. Codon Usage Adaptation in TuMV

**Figure 6:**
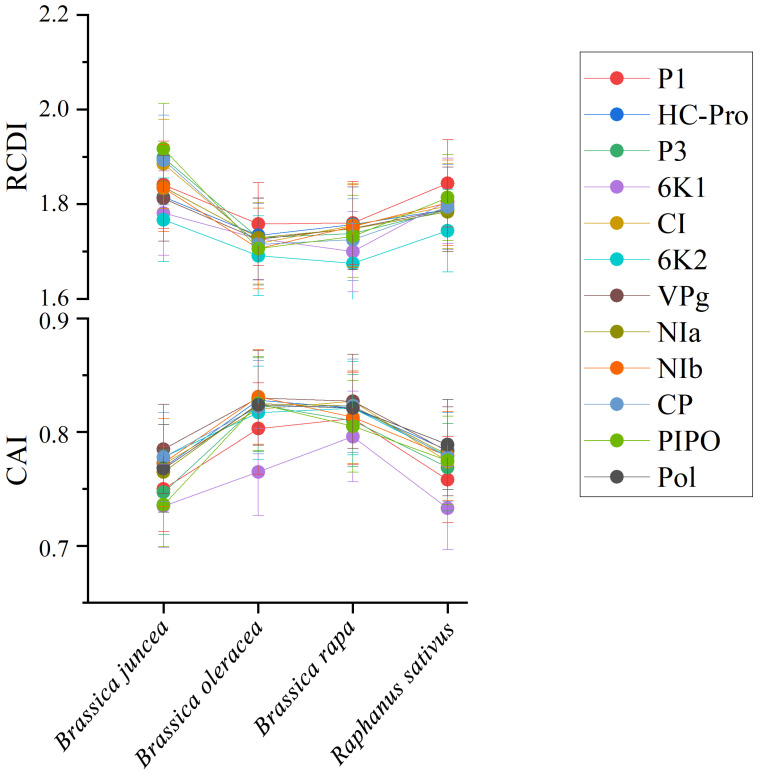
The codon adaptation index (CAI) analysis and relative codon deoptimization index (RCDI) analysis of the eleven protein and complete polyprotein-coding sequences of turnip mosaic virus in relation to the natural hosts. The *x*-axis indicates the sequences isolated from different hosts.

**Figure 7:**
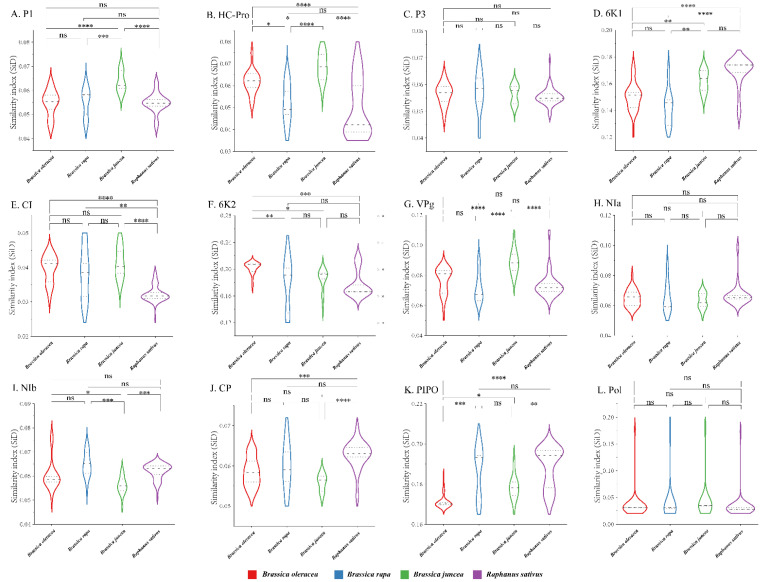
The similarity index (SiD) analysis of the individual protein (**A**–**K**) and complete polyprotein (**L**) coding sequences of turnip mosaic virus in relation to the natural hosts. One-way ANOVA and Tukey’s test were employed to compare the mean of the SiD values pertaining to the different hosts. Asterisk indicated the differential SiD value of turnip mosaic virus between four hosts is statistically significant or very significant (*p* < 0.001 or *p* < 0.0001), “ns”, not significant, *p* > 0.05. * *p* < 0.05; ** *p* < 0.01, *** *p* < 0.001; **** *p* < 0.0001.

To quantify the adaptation and codon usage optimization of TuMV to its hosts, codon adaptation index values were calculated. Normally, genes with higher CAI values are more suitable for the host than those with lower CAI values. The average CAI values of polyprotein sequences were 0.824, 0.821, 0.768, and 0.789 for *B. oleracea, B. rapa, B. juncea,* and *R. sativus* respectively, whereas the highest values for the eleven coding sequences were identified in *B. oleracea* (Figure 6). These results suggest that *B. oleracea* was the most suitable host of TuMV. Additionally, RCDI analysis was conducted to show the cumulative effects of codon bias on a single gene expression. The means of the RCDI values were highest for *B. juncea*, and the lowest RCDI values were observed for *B. oleracea* (Figure 6), indicating that codon usage deoptimization was highest for *B. juncea* and lowest for *B. oleracea*. Then, a SiD analysis was performed to understand how the codon usage patterns of *B. oleracea*, *B. rapa*, *B. juncea*, and *R. sativus* affected the TuMV codon usage pattern (Figure 7). The SiD value among the complete polyproteins is similar for *B. juncea* and *B. oleracea* (Figure 7). In the 11 protein-coding sequences of TuMV, the highest SiD values were observed in *B. oleracea*, *B. juncea*, and *R. sativus* (expect P3). Combining the above CAI and RCDI analysis shows that through TuMV evolution, *B. oleracea* perhaps had a greater impact on the virus than the other hosts from the present data.

## 4. Discussion

TuMV is an important viral disease in vegetable crops, and predecessors also published many reports about vegetable crop virus disease [40,41,42]. Previously, the genetic evolution of TuMV in terms of phylogenetics, dynamics, and migration was effectively performed and based on analyses of complete or partial genome sequences in Europe, the Middle East, East Asia, and Oceania [15,16,17,18,19,20,21,22]. Previous studies have reported that approximately 75% of the isolates from TuMV populations are recombinants [17,43]. Phylogenetic analyses performed by Ohshima et al. (2002), Nguyen et al. (2013), and Kawakubo et al. (2022), which were based on the complete polyprotein sequence, found six divergent evolutionary lineages [15,17]. Recently, Yasaka et al. (2017) and Kawakubo et al. (2021) reported that six lineages were gathered based on TuMV non-recombinant sequences from Europe, the Middle East, East Asia, and Oceania [21].

Codon usage patterns of viruses reflect evolutionary changes, such as adaption, evolution, evasion from host immune systems, and survival [44,45,46,47,48,49,50,51,52]. Now, only limited reports show the codon usage patterns of plant viruses, such as citrus tristeza virus (CTV) [50], rice strape virus (RSV) [51], papaya ringspot virus (PRSV) [52], potato virus M (PVM) [53], sugarcane mosaic virus (SCMV) [54], broad bean wilt virus 2 (BBWV2) [55], rice black-streaked dwarf virus (RBSDV) [56], narcissus degeneration virus (NDV) [57], narcissus late season yellows virus (NLSYV) [57], and narcissus yellow stripe virus (NYSV) [57]. Here, the composition and codon usage patterns of TuMV based on the complete genome were estimated. In general, genomes with AU-rich virus compositions tend to contain codons ending with A and U as opposed to viral genomes with GC-rich compositions, which tend to contain codons that end with G and C [43,46,47,53]. In this study, in comparison, A/C-terminal codons are more strongly preferred in TuMV gene sequences. The nucleotide bias analysis in previous studies showed that the composition constraint was mainly affected by the preferred codon [43,46,47,53]. In the present study, A/G was found to be most abundant in the TuMV protein-coding regions, which supports the presence of mutation pressure.

Low codon usage biases were also observed for RSV, CTV, PRSV, PVM, SCMV, BBWV2, RBSDV, NDV, NLSYV, and NYSV [50,51,52,53,54,55,56,57]. For TuMV, similar lower codon usage patterns were also found with ENC values higher than 35, which indicated a low degree of preference. Additionally, the neutrality plot, ENC-plot, and PR2 analyses showed that the evolution of the TuMV genome has been shaped by mutation and natural selection to varying degrees. Moreover, the neutrality plot and ENC-plot analyses indicated that natural selection is the major factor that induces the codon usage bias of TuMV, which is consistent with PVM and SCMV [53,54].

Previously, several studies have shown that codon usage patterns could affect virus host-specific adaptions [43,46,48,53,54]. In the present study, TuMV and its host adaptions were assessed from the viewpoint of codon usage bias. CAI analysis demonstrated that TuMV genes were more strongly adapted to *B. oleracea* than to *B. juncea, B. rapa,* and *R. sativus*. Furthermore, RCDI analysis showed that strong codon usage deoptimization occurred in *B. oleracea*. Generally, low RCDI values mean strong adaptations to hosts [58]. Thus, both the CAI and RCDI results were consistent. Our SiD analysis indicated that the selection pressure of host plants on TuMV was similar because almost consistent SiD values for isolates were observed from *B. oleracea*, *B. juncea*, *B. rapa*, and *R. sativus* based on polyprotein. However, *B. oleracea*, *B. juncea*, and *R. sativus* showed differences in impacts on the evolution of TuMV 11 protein-coding sequences. It is worth noting that only reference genome sequences of four host species were used in this study. The host population panel is not adequately represented in this study due to only the reference genomes of some species are available in the database. Similarly, during the evolution of the Zika virus (ZIKV), both the CAI and RCDI results showed that the Zika virus (ZIKV) was most strongly adapted to *Aedes aegypti* or *Homo sapiens* while in the SiD analysis, *Ae. albopictus* is potentially the new, preferred vector of ZIKV because the selection pressure exerted by *Ae. albopictus* on codon usage patterns was greater than the selection pressure imposed by *Ae. aegypti* or *H. sapiens* [43].

In conclusion, the detailed codon usage patterns of TuMV were studied for the first time according to complete genome sequences to gain knowledge into the genetic evolution and host adaptability of TuMV. Our study also provides a better understanding of the evolutionary changes of TuMV, which should be considered for the prevention and control of this virus.

## Figures and Tables

**Table 1:** The relative synonymous codon usage (RSCU) value of 59 codons encoding 18 amino acids according to 11 protein and polyprotein of turnip mosaic virus.

Codon	aa	TuMV											
		P1	HC	P3	6K1	CI	6K2	Vpg	Nla	Nlb	CP	PIPO	Pol
TTT	F	0.98	0.66	**1.02**	**1.41**	0.95	0.37	0.79	0.90	0.81	0.91	**1.75**	0.87
TTC	F	**1.02**	**1.34**	0.98	0.59	**1.05**	**1.63**	**1.21**	**1.10**	**1.19**	**1.09**	0.24	**1.13**
TTA	L	0.65	0.72	1.35	0.81	0.62	0.49	0.49	1.04	0.50	**1.32**	2.35	0.80
TTG	L	**1.76**	**1.31**	**1.44**	0.78	0.90	1.16	1.08	**1.31**	**1.41**	1.02	**2.40**	**1.25**
CTT	L	0.66	0.82	0.68	1.00	**1.73**	**1.69**	1.13	0.89	0.89	0.93	0.07	1.03
CTC	L	1.29	0.97	0.68	**2.53**	1.31	0.75	**1.68**	1.08	1.12	1.15	0.01	1.14
CTA	L	0.79	1.23	1.11	0.70	0.98	1.07	0.57	1.26	0.87	0.83	0.44	0.98
CTG	L	0.86	0.95	0.75	0.17	0.46	0.85	1.05	0.42	1.19	0.75	0.74	0.80
ATT	I	**1.22**	0.65	0.87	0.81	0.81	**1.45**	0.85	1.11	0.74	**1.20**	0.26	0.90
ATC	I	1.13	1.12	**1.36**	0.62	1.06	1.11	0.89	0.76	**1.15**	0.84	**1.73**	**1.08**
ATA	I	0.65	**1.23**	0.77	**1.57**	**1.12**	0.44	**1.26**	**1.13**	1.10	0.96	1.01	1.02
GTT	V	1.05	1.11	0.90	0.94	**1.14**	1.31	0.67	0.68	**1.03**	0.90	0.09	1.02
GTC	V	0.94	**1.18**	**1.08**	**1.57**	0.86	0.22	0.47	1.10	1.02	0.66	**2.81**	0.95
GTA	V	0.79	0.70	0.97	0.39	0.90	0.82	0.52	0.67	0.98	1.20	0.32	0.84
GTG	V	**1.23**	1.01	1.05	1.11	1.11	**1.65**	**2.34**	**1.56**	0.97	**1.25**	0.78	**1.19**
TCT	S	0.42	0.56	0.67	1.56	0.78	0.88	1.61	0.49	0.21	1.18	0.47	0.62
TCC	S	0.69	0.51	0.39	1.55	0.49	0.83	0.46	0.71	0.32	1.16	0.37	0.55
TCA	S	1.38	**1.73**	1.06	0.09	**1.53**	1.24	1.07	1.18	**1.78**	0.60	**1.64**	1.38
TCG	S	0.38	0.71	0.50	0.00	0.53	0.23	0.06	0.47	1.27	0.09	1.00	0.57
AGT	S	**1.61**	1.10	**1.88**	1.15	1.20	0.34	0.61	1.34	1.11	**1.61**	1.02	1.35
AGC	S	1.53	1.40	1.50	**1.65**	1.47	**2.48**	**2.19**	**1.81**	1.32	1.36	1.50	**1.52**
CCT	P	0.67	0.44	0.62	2.30	0.62	1.63	0.81	0.42	0.87	0.16	0.00	0.62
CCC	P	0.60	0.32	0.33	1.54	0.34	**2.28**	0.42	0.24	0.38	0.65	0.39	0.43
CCA	P	**2.15**	**2.70**	**2.64**	0.15	**2.45**	0.09	**2.26**	**2.54**	**2.41**	**1.79**	**2.61**	**2.36**
CCG	P	0.58	0.53	0.41	0.00	0.59	0.00	0.51	0.81	0.34	1.40	0.02	0.59
ACT	T	0.66	0.84	1.03	1.33	0.87	0.42	0.54	0.89	0.89	0.65	0.00	0.81
ACC	T	1.33	0.57	0.66	0.25	0.57	1.38	0.74	0.81	0.72	0.76	0.40	0.76
ACA	T	**1.54**	**1.65**	**1.49**	**2.13**	**2.01**	0.76	**2.16**	**1.21**	**1.80**	1.11	**2.22**	**1.67**
ACG	T	0.47	0.93	0.82	0.30	0.55	**1.45**	0.56	1.08	0.59	**1.48**	0.83	0.76
GCT	A	0.76	0.78	0.83	0.29	0.89	0.00	**1.50**	0.77	0.89	0.83	1.21	0.85
GCC	A	0.88	0.45	0.81	0.82	0.61	**0.07**	1.15	0.69	0.30	0.52	0.19	0.64
GCA	A	**1.81**	**2.15**	**1.65**	**2.20**	**1.81**	0.03	0.92	**2.09**	**2.13**	**2.04**	**2.35**	**1.88**
GCG	A	0.55	0.62	0.71	0.69	0.69	0.03	0.43	0.45	0.69	0.61	0.24	0.63
TAT	Y	0.73	0.75	0.63	0.63	**1.07**	0.00	0.75	0.90	**1.13**	0.61	0.58	0.88
TAC	Y	**1.27**	**1.25**	**1.37**	**1.37**	0.93	0.00	**1.25**	**1.10**	0.87	**1.39**	**1.42**	**1.12**
CAT	H	0.68	0.94	**1.17**	**1.31**	0.79	0.44	0.67	0.00	0.60	**1.24**	0.00	0.87
CAC	H	**1.32**	**1.06**	0.83	0.69	**1.21**	**1.56**	**1.33**	**0.97**	**1.40**	0.76	0.00	**1.13**
CAA	Q	0.95	**1.02**	**1.24**	**1.53**	0.85	**1.02**	0.88	**1.33**	**1.03**	0.80	**1.33**	**1.02**
CAG	Q	**1.05**	0.98	0.76	0.47	**1.15**	0.97	**1.12**	0.00	0.97	**1.20**	0.67	0.98
AAT	N	0.53	0.79	**1.11**	0.20	0.91	0.82	0.90	0.69	0.76	0.83	0.03	0.83
AAC	N	**1.47**	**1.21**	0.89	**1.76**	**1.09**	**1.18**	**1.10**	**1.31**	**1.24**	**1.17**	**1.69**	**1.17**
AAA	K	0.73	0.97	0.97	0.60	**1.02**	**1.09**	0.83	**1.11**	0.91	0.89	**1.31**	0.92
AAG	K	**1.27**	**1.03**	**1.03**	**1.40**	0.98	0.91	**1.17**	0.89	**1.09**	**1.11**	0.69	1.08
GAT	D	0.55	**1.10**	**1.23**	**1.14**	**1.02**	0.83	**1.08**	**1.02**	**1.19**	0.92	**1.21**	**1.05**
GAC	D	**1.45**	0.90	0.77	0.86	0.98	**1.17**	0.92	0.98	0.81	**1.08**	0.79	0.95
GAA	E	0.94	0.92	**1.08**	**1.23**	0.94	**1.80**	**1.20**	0.70	**1.18**	0.90	**1.15**	**1.01**
GAG	E	**1.06**	**1.08**	0.92	0.77	**1.06**	0.20	0.80	**1.30**	0.82	**1.10**	0.85	0.99
TGT	C	0.33	**1.08**	**1.36**	**1.64**	**1.24**	**1.77**	**1.79**	**1.16**	**1.01**	0.00	**1.96**	**1.12**
TGC	C	**1.66**	0.92	0.64	0.36	0.76	0.23	0.21	0.84	0.99	**2.00**	0.03	0.88
CGT	R	0.59	0.53	0.41	1.08	0.53	1.07	0.85	0.56	0.36	1.19	0.34	0.63
CGC	R	0.86	1.33	1.10	0.70	0.37	0.49	0.50	0.21	1.03	0.75	1.48	0.77
CGA	R	0.75	0.71	0.53	**1.52**	1.17	**2.39**	1.12	1.56	**1.90**	1.15	0.70	1.09
CGG	R	0.21	0.32	0.10	0.62	0.44	1.21	0.13	1.14	0.72	0.43	0.12	0.41
AGA	R	**2.07**	**1.81**	**2.30**	1.43	**2.44**	0.48	1.66	**1.85**	1.23	**1.43**	**3.24**	**1.90**
AGG	R	1.52	1.29	1.56	0.65	1.05	0.36	**1.74**	0.69	0.77	1.06	0.13	1.20
GGT	G	0.66	0.95	1.11	0.11	1.07	0.74	1.03	**1.25**	0.95	**1.31**	0.48	1.01
GGC	G	0.63	1.15	**1.33**	0.02	0.67	0.83	0.80	0.74	0.72	1.23	**3.52**	0.86
GGA	G	**1.89**	**1.40**	1.23	**3.41**	**1.53**	**2.34**	**1.72**	1.24	**1.67**	1.18	0.01	**1.54**
GGG	G	0.82	0.50	0.32	0.46	0.73	0.08	0.45	0.77	0.66	0.27	1.75	0.59

The most frequently used codons are shown in bold.

## Data Availability

The Tables S1–S3 in supplementary file.

## Supplementary Materials

The following supporting information can be downloaded at: https://www.mdpi.com/article/10.3390/v14102267/s1. Figure S1: Neighbor-Joining (NJ) trees calculated from the polyprotein sequences of non-recombinant turnip mosaic virus. Numbers at each node indicate the percentage of bootstrap samples in the NJ trees. Horizontal branch lengths are drawn to scale, with the bar indicating 0.1 nt replacements per site; Figure S2: Relative and cumulative inertia of the 35 axes from a COA of the RSCU values based on the individual protein (A–K) and complete polyprotein (L) coding sequences of turnip mosaic virus; Table S1: The turnip mosaic virus isolates used in this study; Table S2: The detail of codons in the individual protein encoding regions of turnip mosaic virus; Table S3: The relative synonymous codon usage (RSCU) value of 59 codons of 18 amino acids of turnip mosaic virus polyprotein and the RSCU value of different hosts.
