# Supplementary material for: Host Plants Shape the Codon Usage Pattern of Turnip Mosaic Virus
Source: Viruses. 2022 Oct 15;14(10):2267. doi: 10.3390/v14102267 (PMC9607058; doi:10.3390/v14102267)
Supplement: Supplementary file 1 [file viruses-14-02267-s001.zip › Table.S1.pdf]

Table S1. The turnip mosaic virus isolates used in this study.

Accesion number	Isolate	Host	Country	Years	Reference
KY111274	DJ1	*Raphanus sativus*	SouthKorea	2015	Gong et al., 2016
KY111273	DJ2	*R.sativus*	SouthKorea	2015	Gong et al., 2016
KY111272	DJ3	*R.sativus*	SouthKorea	2015	Gong et al., 2016
KY111271	DJ4	*R.sativus*	SouthKorea	2015	Gong et al., 2016
KY111270	DJ5	*R.sativus*	SouthKorea	2015	Gong et al., 2016
KY111269	DJ6	*R.sativus*	SouthKorea	2015	Gong et al., 2016
KY111268	SW1	*R.sativus*	SouthKorea	2015	Gong et al., 2016
KX674733	GJS4	*R.sativus*	SouthKorea	2015	Gong et al., 2016
KX674732	GJS3	*R.sativus*	SouthKorea	2015	Gong et al., 2016
KX674731	GJS2	*R.sativus*	SouthKorea	2015	Gong et al., 2016
KX674730	KIH2	*R.sativus*	SouthKorea	2015	Gong et al., 2016
KX674729	KIH1	*R.sativus*	SouthKorea	2015	Gong et al., 2016
KX579484	Jj16	*R.sativus*	SouthKorea	2014	Chung et al., 2016
DQ648591	CAR37A	*R.sativus*	Poland	2006	Kozubek et al., 2006
KU140422	R065	*R.sativus*	SouthKorea	2014	Chung et al., 2015
KU140421	R041	*R.sativus*	SouthKorea	2014	Chung et al., 2015
KR153040	WFLB14	*R.sativus*	China	2014	Chung et al., 2015
KR153039	LWLB	*R.sativus*	China	2014	Chung et al., 2015
KR153038	CCLB	*B.oleracea*	China	2014	Chung et al., 2015
HQ637383	CAR51	*R.sativus*	Poland	2004	Chung et al., 2015
AB252117	GRC42	*R.sativus*	Greece	1999	Ohshima et al., 2007
AB093611	BZ1	*R.sativus*	Brazil	1996	Tomimura et al., 2003
AB093606	RUS1	*R.sativus*	Russia	1993	Tomimura et al., 2003
AB093598	Al	*B.oleracea*	Italy	1968	Tomimura et al., 2003
AP017884	TUR77	*B.oleracea*	Turkey	2007	Yasaka et al., 2017
AP017854	IRNWRSh40	*B.oleracea*	Iran	2007	Yasaka et al., 2017
AP017846	IRNSRSh35	*B.rapa*	Iran	2007	Yasaka et al., 2017
AP017845	IRNRRSh35	*Sisymbrium loeselii*	Iran	2007	Yasaka et al., 2017
AP017840	GRC39	*S.loeselii*	Greece	1996	Yasaka et al., 2017
AP017839	GRC38	*S.loeselii*	Greece	1996	Yasaka et al., 2017
AP017819	GRC5	*S.loeselii*	Greece	1993	Yasaka et al., 2017
AP017814	TUR79	*S.loeselii*	Turkey	2007	Yasaka et al., 2017
AP017808	IRNTuSh18	*S.loeselii*	Iran	2007	Yasaka et al., 2017
AP017801	IRNTOFS6	*Rapistrum rugosum*	Iran	2006	Yasaka et al., 2017
AP017800	IRNTOFS3	*R.rugosum*	Iran	2006	Yasaka et al., 2017
AP017799	IRNTOFS2	*R.rugosum*	Iran	2006	Yasaka et al., 2017
AP017797	IRNTKE	*R.rugosum*	Iran	2006	Yasaka et al., 2017
AP017796	IRNTlm1	*B.rapa*	Iran	2004	Yasaka et al., 2017
AP017790	IRNS4	*S.loeselii*	Iran	2003	Yasaka et al., 2017
AP017789	IRNS3	*S.loeselii*	Iran	2003	Yasaka et al., 2017
AP017788	IRNS1	*B.oleracea*	Iran	2003	Yasaka et al., 2017
AP017785	IRNRN6	Orchis	Iran	2005	Yasaka et al., 2017
AP017784	IRNRkaraj	Orchis	Iran	2004	Yasaka et al., 2017
AP017783	IRNRK	Orchis	Iran	2004	Yasaka et al., 2017
AP017780	IRNRaNi3	Orchis	Iran	2005	Yasaka et al., 2017
AP017779	IRNRaFCh	*B.rapa*	Iran	2006	Yasaka et al., 2017
AP017778	IRNMY57	*R.rugosum*	Iran	2004	Yasaka et al., 2017
AP017769	IRNKBS65	*R.rugosum*	Iran	2006	Yasaka et al., 2017
AP017768	IRNKBS58	*B.oleracea*	Iran	2006	Yasaka et al., 2017
AP017756	GRC43	*S.loeselii*	Greece	1999	Yasaka et al., 2017

AP017754	IRNEAM1	*B.rapa*	Iran	2005	Yasaka et al., 2017
AB701717	GBR83	*R.sativus*	UK	2002	Nguyen et al., 2013
AB701698	BEL1	*R.sativus*	Belgium	1986	Nguyen et al., 2013
AB701697	ASP	*B.rapa*	Germany	1995	Nguyen et al., 2013
AB701693	OS	*R.sativus*	Germany	1981	Nguyen et al., 2013
AB701692	ORM	*R.sativus*	Germany	1983	Nguyen et al., 2013
AB701690	OM-N	*R.sativus*	Germany	1981	Nguyen et al., 2013
AB701691	OM-A	*R.sativus*	Germany	1981	Nguyen et al., 2013
AP017815	TUR242	*R.sativus*	Turkey	2012	Yasaka et al., 2017
AP017804	IRNTRT9	*B.rapa*	Tehran	2006	Yasaka et al., 2017
AB440238	IRNTRa6	*B.rapa*	Tehran	2004	Farzadfar et al., 2009
AP017803	IRNTRa9	*B.oleracea*	Tehran	2004	Yasaka et al., 2017
AP017770	IRN KhCa	*B.oleracea*	Tehran	2005	Yasaka et al., 2017
AP017763	IRNDM	*B.oleracea*	Iran	2004	Yasaka et al., 2017
AP017794	IRNST	*R.sativus*	Tehran	2005	Yasaka et al., 2017
AP017773	IRNMB6	*B.rapa*	Iran	2003	Yasaka et al., 2017
AP017795	IRNTH	*Brassica juncea*	Tehran	2006	Yasaka et al., 2017
AP017873	TUR52	*B.juncea*	Turkey	2006	Yasaka et al., 2017
AB362513	TUR9	*B.juncea*	Turkey	2005	Korkmaz et al., 2008
AP017864	TUR10	*R.sativus*	Turkey	2006	Yasaka et al., 2017
LC537519	CHSE1AB	*B.juncea*	China	1999	Kawakubo et al., 2021
LC537528	CHZJ23	*B.juncea*	China	1999	Kawakubo et al., 2021
LC537525	CHZH36	*B.juncea*	China	2000	Kawakubo et al., 2021
AB701725	ITA8	*B.juncea*	Italy	1993	Nguyen et al., 2013
AB093600	ITA7	*B.oleracea*	Italy	1990	Tomimura et al., 2003
KY111267	SW2	*B.oleracea*	Not known	2015	Kawakubo et al., 2021
LC537507	CHN305	*B.oleracea*	China	2014	Kawakubo et al., 2021
KX579485	Jj13	*B.oleracea*	Not known	2014	Kawakubo et al., 2021
LC537496	CHN172	*B.oleracea*	China	2011	Kawakubo et al., 2021
AB701703	DNK3	*B.oleracea*	Not known	1978	Nguyen et al., 2013
AB252107	CZE5	*B.oleracea*	South Bohemia	1993	Ohshima et al., 2007
AP017867	TUR20	*B.oleracea*	Turkey	2006	Yasaka et al., 2017
LC537585	UKR28	*B.oleracea*	Ukraine	2014	Kawakubo et al., 2021
LC537591	UKR47	*B.oleracea*	Ukraine	2014	Kawakubo et al., 2021
LC537592	UKR49	*B.oleracea*	Ukraine	2014	Kawakubo et al., 2021
LC537590	UKR46	*B.oleracea*	Ukraine	2014	Kawakubo et al., 2021
LC537563	MYA1	*B.oleracea*	Myanmar	2013	Kawakubo et al., 2021
LC537521	CHYK56A	*B.oleracea*	China	2000	Kawakubo et al., 2021
LC537520	CHYK20A	*B.oleracea*	China	2000	Kawakubo et al., 2021
LC537500	CHN229	*B.oleracea*	China	2012	Kawakubo et al., 2021
LC537501	CHN241	*B.oleracea*	China	2012	Kawakubo et al., 2021
LC537503	CHN244	*B.oleracea*	China	2012	Kawakubo et al., 2021
LC537527	CHZH34A	*B.oleracea*	China	2000	Kawakubo et al., 2021
LC537526	CHZH33A	*B.oleracea*	China	2000	Kawakubo et al., 2021
AB747293	VIET80	*B.oleracea*	Vietnam	2007	Nguyen et al., 2013
AB747306	VIET170	*B.juncea*	Vietnam	2007	Nguyen et al., 2013
AB747312	VIET177	*B.juncea*	Vietnam	2008	Nguyen et al., 2013
AB747299	VIET158	*B.juncea*	Vietnam	2007	Nguyen et al., 2013
AB747307	VIET172	*B.juncea*	Vietnam	2007	Nguyen et al., 2013
LC537541	CZE11	*B.rapa*	The Czech Republic	1994	Kawakubo et al., 2021
LC537542	CZE12	*B.juncea*	The Czech Republic	1994	Kawakubo et al., 2021
LC537545	CZE15	*R.sativus*	The Czech Republic	1994	Kawakubo et al., 2021
LC537543	CZE13	*R.sativus*	The Czech Republic	1994	Kawakubo et al., 2021
LC537547	CZE17	*R.sativus*	The Czech Republic	1994	Kawakubo et al., 2021
LC537544	CZE14	*R.sativus*	The Czech Republic	1994	Kawakubo et al., 2021
LC537546	CZE16	*R.sativus*	The Czech Republic	1994	Kawakubo et al., 2021
LC537537	CZE2	*R.sativus*	The Czech Republic	1993	Kawakubo et al., 2021
AP017837	GRC34	*B.juncea*	Greece	1994	Yasaka et al., 2017

AP017838	GRC35	*B.juncea*	Greece	1993	Yasaka et al., 2017
AP017823	GRC12	*R.sativus*	Greece	1993	Yasaka et al., 2017
AP017824	GRC16	*B.oleracea*	Greece	1993	Yasaka et al., 2017
AP017830	GRC26	*B.oleracea*	Greece	1993	Yasaka et al., 2017
AP017820	GRC6	*B.oleracea*	Greece	1993	Yasaka et al., 2017
AP017818	GRC4	*R.sativus*	Greece	1993	Yasaka et al., 2017
AB989637	AUST21	*R.sativus*	Australia	2011	Yasaka et al., 2015
AP017821	GRC10	*B.juncea*	Greece	1993	Yasaka et al., 2017
AP017827	GRC23	*B.juncea*	Greece	1993	Yasaka et al., 2017
AP017828	GRC24	*B.juncea*	Greece	1993	Yasaka et al., 2017
AP017816	GRC2	*B.juncea*	Greece	1993	Yasaka et al., 2017
AP017817	GRC3	*B.juncea*	Greece	1993	Yasaka et al., 2017
AP017822	GRC11	*R.sativus*	Greece	1993	Yasaka et al., 2017
AP017829	GRC25	*R.sativus*	Greece	1993	Yasaka et al., 2017
LC537477	CHHH29A	*R.sativus*	China	1999	Kawakubo et al., 2021
AB747297	VIET138	*R.sativus*	Vietnam	2007	Nguyen et al., 2017
AB747313	VIET178	*R.sativus*	Vietnam	2008	Nguyen et al., 2017
AB747315	VIET180	*R.sativus*	Vietnam	2008	Nguyen et al., 2017
AB747314	VIET179	*R.sativus*	Vietnam	2008	Nguyen et al., 2017
LC537481	CHK55A	*R.sativus*	Vietnam	2000	Kawakubo et al., 2021
AB747298	VIET153	*R.sativus*	Vietnam	2007	Nguyen et al., 2017
LC537578	THA5	*R.sativus*	Thailand	2014	Kawakubo et al., 2021
LC537577	THA3	*R.sativus*	Thailand	2014	Kawakubo et al., 2021
LC537579	THA7	*R.sativus*	Thailand	2014	Kawakubo et al., 2021
LC537576	THA2	*R.sativus*	Thailand	2014	Kawakubo et al., 2021
LC537575	THA1	*R.sativus*	Thailand	2014	Kawakubo et al., 2021
LC537582	THA13	*R.sativus*	Thailand	2014	Kawakubo et al., 2021
LC537581	THA9	*R.sativus*	Thailand	2014	Kawakubo et al., 2021
LC537580	THA8	*R.sativus*	Thailand	2014	Kawakubo et al., 2021
LC537512	CHN328	*R.sativus*	China	2014	Kawakubo et al., 2021
LC537510	CHN322	*B.oleracea*	China	2014	Kawakubo et al., 2021
LC537506	CHN301	*R.sativus*	China	2014	Kawakubo et al., 2021
KF246570	ZH1	*R.sativus*	China	2012	Peng et al., 2013
KC119185	BJ-B01	*R.sativus*	China	2010	Peng et al., 2013
KC119186	BJ-B02	*R.sativus*	Chinan	2010	Peng et al., 2013
KC119187	BJ-B03	*B.oleracea*	China	2010	Peng et al., 2013
LC537530	CHZJ27A	*B.oleracea*	China	1999	Kawakubo et al., 2021
LC537534	SAS1	*B.oleracea*	China	1997	Kawakubo et al., 2021
LC537529	CHZJ25A	*B.rapa*	China	1999	Kawakubo et al., 2021
LC537479	CHHZ3A	*S.loeselii*	China	2000	Kawakubo et al., 2021
AB747287	VIET56	*S.loeselii*	Moc Chau	2007	Nguyen et al., 2013
AB747288	VIET58	*S.loeselii*	Moc Chau	2007	Nguyen et al., 2013
AB747308	VIET173	*S.loeselii*	Bac Giang	2007	Nguyen et al., 2013
AB747309	VIET174	*S.loeselii*	Bac Giang	2007	Nguyen et al., 2013
AB747310	VIET175	*S.loeselii*	Bac Giang	2007	Nguyen et al., 2013
LC639370	AD886J	*R.sativus*	Japan	2002	Kawakubo et al., 2022
LC639371	AD887J	*R.sativus*	Japan	2002	Kawakubo et al., 2022
LC639372	AD888J	*R.sativus*	Japan	2002	Kawakubo et al., 2022
LC639394	FB999J	*B.rapa*	Japan	2004	Kawakubo et al., 2022
LC639396	FD810J	*R.sativus*	Japan	2000	Kawakubo et al., 2022
LC639397	FD891J	*R.sativus*	Japan	2007	Kawakubo et al., 2022
LC639471	KD302J	*R.sativus*	Japan	2006	Kawakubo et al., 2022
LC639478	KGB394J	*B.oleracea*	Japan	2007	Kawakubo et al., 2022
LC639481	KGB964J	*B.rapa*	Japan	2002	Kawakubo et al., 2022
LC639482	KGD373J	*R.sativus*	Japan	2007	Kawakubo et al., 2022
LC639483	KGD55J	*R.sativus*	Japan	1998	Kawakubo et al., 2022
LC639498	KGD963J	*R.sativus*	Japan	2002	Kawakubo et al., 2022
LC639518	MB649J	*B.rapa*	Japan	2007	Kawakubo et al., 2022
LC639523	MD644J	*R.sativus*	Japan	2007	Kawakubo et al., 2022

LC639525	MD652J	*R.sativus*	Japan	2007	Kawakubo et al., 2022
LC639533	N214	*B.rapa*	Japan	1961	Kawakubo et al., 2022
LC639536	N218	*B.rapa*	Japan	1963	Kawakubo et al., 2022
LC639538	N220	*B.rapa*	Japan	1974	Kawakubo et al., 2022
LC639539	N223	*B.rapa*	Japan	1965	Kawakubo et al., 2022
LC639541	N227	*B.rapa*	Japan	1960	Kawakubo et al., 2022
LC639546	ND260J	*R.sativus*	Japan	2001	Kawakubo et al., 2022
LC639547	ND264J	*R.sativus*	Japan	2001	Kawakubo et al., 2022
LC639548	ND271J	*R.sativus*	Japan	2009	Kawakubo et al., 2022
LC639549	ND276J	*R.sativus*	Japan	2010	Kawakubo et al., 2022
LC639575	OB284J	*Brassica sp.*	Japan	2007	Kawakubo et al., 2022
LC639577	OB995J	*Brassica sp.*	Japan	2001	Kawakubo et al., 2022
LC639585	OKB634J	*Nasturtium officinale*	Japan	2005	Kawakubo et al., 2022
LC639601	OKD627J	*R.sativus*	Japan	2002	Kawakubo et al., 2022
LC639610	ON16J	*B.napus*	Japan	1998	Kawakubo et al., 2022
LC639634	SMD213J	*R.sativus*	Japan	2005	Kawakubo et al., 2022
LC639650	TRD054J	*R.sativus*	Japan	2000	Kawakubo et al., 2022
