# Supplementary material for: Host Plants Shape the Codon Usage Pattern of Turnip Mosaic Virus
Source: Viruses. 2022 Oct 15;14(10):2267. doi: 10.3390/v14102267 (PMC9607058; doi:10.3390/v14102267)
Supplement: Supplementary file 1 [file viruses-14-02267-s001.zip › Table.S3.pdf]

Table S3. The relative synonymous codon usage (RSCU) value of 59 codons of 18 amino acids of turnip mosaic virus polyprotein and the RSCU value of different hosts.

.Codon	aa	TuMV-polyprotein											
		Brassica juncea	Pol-*B. juncea*	*Brassica oleracea*	Pol-*B. oleracea*	*Brassica rapa*	Pol-*B. rapa*	*Raphanus sativus*	Pol-*R. sativus*	*Rapistrum rugosum*	Pol-*R. rugosum*	*Sisymbrium loeselii*	Pol-*S. loeselii*
TTT	F	**1.06***	0.88	0.93	0.83	0.93	0.90	0.91	0.85	**1.31**	0.98	0.85	0.93
TTC	F	0.94	**1.12**	**1.06**	**1.17**	**1.06**	**1.10**	**1.08**	**1.16**	0.69	**1.02**	**1.15**	**1.07**
TTA	L	1.02	0.75	0.68	0.71	0.68	0.82	0.64	0.74	**1.86**	1.05	0.61	1.08
TTG	L	**1.22**	**1.28**	1.26	**1.28**	1.26	**1.26**	1.27	**1.24**	1.09	**1.15**	**1.26**	**1.25**
CTT	L	1.22	1.02	**1.42**	0.94	**1.43**	1.01	**1.37**	1.06	1.37	1.12	1.07	1.05
CTC	L	0.96	1.12	1.23	1.19	1.25	1.15	1.28	1.12	0.56	1.05	1.08	1.12
CTA	L	0.83	1.05	0.62	1.05	0.62	1.00	0.6	1.03	0.72	0.99	1.16	0.88
CTG	L	0.75	0.78	0.78	0.83	0.75	0.77	0.82	0.81	0.4	0.65	0.82	0.62
ATT	I	**1.19**	0.91	1.05	0.87	1.06	0.92	1.02	0.88	**1.43**	1.00	1.01	1.03
ATC	I	0.96	**1.05**	**1.2**	**1.09**	**1.19**	**1.06**	**1.23**	**1.11**	0.7	0.90	**1.73**	0.90
ATA	I	0.85	**1.05**	0.74	1.04	0.74	1.02	0.74	1.01	0.87	**1.10**	0.26	**1.08**
GTT	V	**1.23**	**1.10**	**1.49**	1.04	**1.48**	1.05	**1.46**	1.01	**1.49**	0.99	**1.82**	1.06
GTC	V	0.79	0.93	0.86	0.96	0.85	0.94	0.88	0.94	0.56	0.94	0.59	0.89
GTA	V	0.97	0.88	0.54	0.81	0.53	0.85	0.53	0.87	1.25	0.88	0.95	0.88
GTG	V	0.96	1.09	1.11	**1.19**	1.15	**1.15**	1.13	**1.18**	0.7	**1.19**	0.64	**1.16**
TCT	S	**1.41**	0.61	**1.59**	0.58	**1.59**	0.62	**1.56**	0.62	**1.61**	0.73	**1.55**	0.68
TCC	S	0.98	0.52	0.89	0.53	0.88	0.56	0.9	0.56	0.94	0.50	1.21	0.51
TCA	S	1.05	1.37	1.13	1.30	1.15	1.37	1.1	1.37	1.12	**1.49**	0.58	**1.45**
TCG	S	0.71	0.59	0.66	0.63	0.65	0.59	0.69	0.56	0.6	0.50	1.16	0.57
AGT	S	1.06	1.34	0.85	1.37	0.83	1.32	0.85	1.38	1.23	1.40	0.62	1.41
AGC	S	0.79	**1.57**	0.89	**1.59**	0.89	**1.54**	0.91	**1.51**	0.5	1.38	0.88	1.39
CCT	P	**1.33**	0.60	**1.5**	0.60	**1.53**	0.61	**1.53**	0.63	**1.45**	0.53	**2.02**	0.55
CCC	P	0.8	0.38	0.56	0.36	0.54	0.44	0.53	0.43	0.62	0.51	0.84	0.50
CCA	P	1.16	**2.47**	1.19	**2.54**	1.18	**2.36**	1.18	**2.37**	1.19	**2.37**	0.8	**2.30**
CCG	P	0.72	0.55	0.73	0.50	0.73	0.59	0.75	0.58	0.6	0.59	0.34	0.65
ACT	T	**1.34**	0.84	**1.23**	0.82	**1.25**	0.80	**1.27**	0.81	**1.52**	0.81	**1.94**	0.81
ACC	T	0.95	0.72	0.9	0.73	0.87	0.75	0.89	0.77	0.86	0.75	1.12	0.72
ACA	T	0.98	**1.66**	1.14	**1.68**	1.13	**1.72**	1.08	**1.63**	1.09	**1.75**	0.53	**1.77**
ACG	T	0.73	0.78	0.73	0.76	0.74	0.73	0.76	0.80	0.54	0.69	0.41	0.70
GCT	A	**1.5**	0.87	**1.67**	0.84	**1.68**	0.88	**1.65**	0.82	**1.81**	0.93	**1.26**	0.90
GCC	A	0.97	0.58	0.7	0.64	0.69	0.62	0.72	0.64	0.69	0.56	0.95	0.59
GCA	A	0.96	**1.90**	0.99	**1.86**	0.99	**1.88**	0.98	**1.89**	1.06	**1.99**	1.07	**1.94**
GCG	A	0.51	0.65	0.64	0.66	0.64	0.63	0.65	0.65	0.44	0.52	0.71	0.58
TAT	Y	**1.23**	0.88	0.9	0.84	0.9	0.93	0.87	0.89	**1.48**	0.96	**1.03**	0.89
TAC	Y	0.73	**1.12**	**1.07**	**1.16**	**1.07**	**1.07**	**1.1**	**1.11**	0.47	**1.04**	0.97	**1.11**
CAT	H	**1.22**	0.86	**1.09**	0.85	**1.08**	0.87	**1.05**	0.88	**1.28**	0.89	0.84	0.93

CAC	H	0.66	**1.14**	0.86	**1.15**	0.87	**1.13**	0.91	**1.12**	0.63	**1.11**	**1.17**	**1.07**
CAA	Q	**1.21**	0.91	**1.03**	0.87	**1.02**	**1.01**	0.98	0.98	**1.49**	**1.22**	**1.04**	**1.22**
CAG	Q	0.74	**1.09**	0.96	**1.13**	0.97	0.99	**1.01**	**1.02**	0.5	0.78	0.96	0.78
AAT	N	**1.22**	0.78	0.86	0.75	0.84	0.82	0.83	0.81	**1.32**	0.95	0.58	0.97
AAC	N	0.75	**1.22**	**1.13**	**1.25**	**1.15**	**1.18**	**1.16**	**1.19**	0.65	**1.05**	**1.42**	**1.03**
AAA	K	**1.13**	0.89	0.89	0.87	0.87	0.91	0.89	0.91	**1.41**	**1.00**	**1.11**	0.97
AAG	K	0.87	**1.11**	**1.11**	**1.13**	**1.12**	**1.09**	**1.1**	**1.09**	0.56	**1.00**	0.89	**1.03**
GAT	D	**1.31**	**1.09**	**1.23**	**1.04**	**1.23**	**1.07**	**1.22**	**1.04**	**1.45**	**1.14**	**1.39**	**1.11**
GAC	D	0.69	0.91	0.76	0.96	0.77	0.93	0.78	0.96	0.55	0.86	0.61	0.89
GAA	E	**1.13**	0.94	0.93	0.95	0.92	**1.05**	0.92	0.96	**1.45**	**1.25**	**1.17**	**1.22**
GAG	E	0.83	**1.06**	**1.07**	**1.05**	**1.08**	0.95	**1.08**	**1.04**	0.55	0.75	0.83	0.78
TGT	C	**1**	**1.13**	**1.02**	**1.16**	**1.03**	**1.15**	**1.01**	**1.12**	**1.27**	**1.10**	**1.18**	**1.06**
TGC	C	0.89	0.87	0.9	0.84	0.89	0.85	0.89	0.88	0.54	0.90	0.82	0.94
CGT	R	0.9	0.61	0.95	0.64	0.97	0.61	0.93	0.67	1.49	0.72	**1.61**	0.67
CGC	R	0.61	0.76	0.53	0.74	0.51	0.78	0.52	0.78	0.53	0.70	0.64	0.67
CGA	R	1.02	1.09	0.61	1.05	0.59	1.09	0.57	1.08	1.31	1.11	1.39	1.14
CGG	R	0.73	0.46	0.51	0.46	0.5	0.41	0.49	0.43	0.48	0.34	0.24	0.29
AGA	R	**1.6**	**1.92**	**2.01**	**2.03**	**2.04**	**1.94**	**2.07**	**1.88**	**1.54**	**2.02**	1.12	**2.06**
AGG	R	1.14	1.16	1.38	1.09	1.38	1.16	1.41	1.16	0.66	1.11	1	1.18
GGT	G	1.17	1.06	1.23	1.02	1.25	1.05	1.25	1.01	1.28	1.09	1.25	1.04
GGC	G	0.64	0.77	0.65	0.79	0.64	0.81	0.64	0.88	0.42	0.80	0.32	0.85
GGA	G	**1.29**	**1.56**	**1.39**	**1.63**	**1.4**	**1.56**	**1.38**	**1.51**	**1.55**	**1.57**	**1.53**	**1.59**
GGG	G	0.85	0.61	0.72	0.56	0.71	0.57	0.73	0.60	0.74	0.54	0.9	0.52

* The most frequently used codons are shown in bold
