# Supplementary figures and images for: Host Plants Shape the Codon Usage Pattern of Turnip Mosaic Virus
Source: Viruses. 2022 Oct 15;14(10):2267. doi: 10.3390/v14102267 (PMC9607058; doi:10.3390/v14102267)

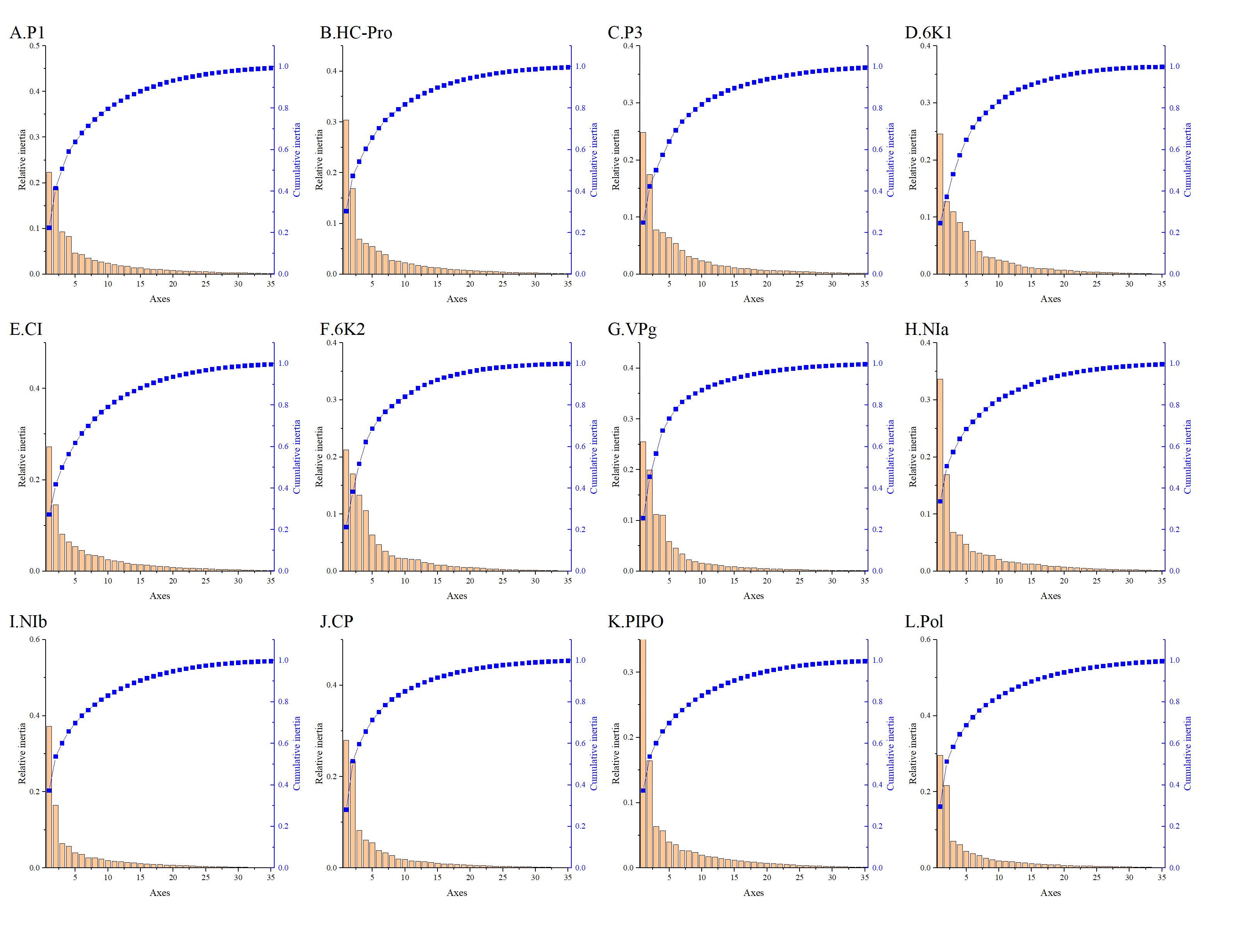

Supplement: Supplementary file 1 [file viruses-14-02267-s001.zip › Fig.S2.tif]
